# Using the Fatigue Severity Scale to inform healthcare decision-making in multiple sclerosis: mapping to three quality-adjusted life-year measures (EQ-5D-3L, SF-6D, MSIS-8D)

**DOI:** 10.1186/s12955-019-1205-y

**Published:** 2019-08-05

**Authors:** E. Goodwin, A. Hawton, C. Green

**Affiliations:** 1 Health Economics Group, Institute of Health Research, University of Exeter Medical School, University of Exeter, Exeter, UK; 2 South West Collaboration for Leadership in Applied Health Research and Care (CLAHRC), University of Exeter Medical School, University of Exeter, Exeter, UK

**Keywords:** Cost effectiveness, Decision making, Multiple sclerosis, Outcomes research, Quality of life, Fatigue

## Abstract

**Background:**

Fatigue has a major influence on the quality of life of people with multiple sclerosis. The Fatigue Severity Scale is a frequently used patient-reported measure of fatigue impact, but does not generate the health state utility values required to inform cost-effectiveness analysis, limiting its applicability within decision-making contexts. The objective of this study was to use statistical mapping methods to convert Fatigue Severity Scale scores to health state utility values from three preference-based measures: the EQ-5D-3L, SF-6D and Multiple Sclerosis Impact Scale-8D.

**Methods:**

The relationships between the measures were estimated through regression analysis using cohort data from 1056 people with multiple sclerosis in South West England. Estimation errors were assessed and predictive performance of the best models as tested in a separate sample (*n* = 352).

**Results:**

For the EQ-5D and the Multiple Sclerosis Impact Scale-8D, the best performing models used a censored least absolute deviation specification, with Fatigue Severity Scale total score, age and gender as predictors. For the SF-6D, the best performing model used an ordinary least squares specification, with Fatigue Severity Scale total score as the only predictor.

**Conclusions:**

Here we present algorithms to convert Fatigue Severity Scales scores to health state utility values based on three preference-based measures. These values may be used to estimate quality-adjusted life-years for use in cost-effectiveness analyses and to consider the health-related quality of life of people with multiple sclerosis, thereby informing health policy decisions.

**Electronic supplementary material:**

The online version of this article (10.1186/s12955-019-1205-y) contains supplementary material, which is available to authorized users.

## Background

Over the last two decades, various disease-modifying and symptomatic treatments have been developed for people with Multiple Sclerosis (MS). Meanwhile, increasing emphasis has been placed on achieving “value for money” within healthcare systems [1]. Clinical trials of interventions that target particular symptoms frequently use symptom-specific outcome measures in order to maximise sensitivity and responsiveness to change. Fatigue is the most common symptom experienced by people with MS, and has a considerable impact on quality of life [2]. The Fatigue Severity Scale (FSS) [3] is frequently used in clinical trials of interventions for fatigue in people with MS, including carnitine, amantadine, aspirin, modafinil and cognitive behavioural therapy [4–7]. Symptom-specific outcome measures, such as the FSS, provide a standardised means of describing “health states” that may be experienced by patients, but do not provide data in the format required by many decision-making bodies to assess cost-effectiveness [1].

The quality-adjusted life-year (QALY) is recommended for use as an outcome measure for cost-effectiveness analyses by several national decision-making bodies, eg the National Institute for Health and Care Excellence (NICE) [8–10]. QALYs combine quantity and quality of life in a single measure, by adjusting the number of life-years lived according to the quality-of-life experienced during those years [1]. In order to estimate QALYs, numerical values must be assigned to reflect the quality of life experienced when living in particular health states. These values are commonly obtained using preference-based measures (PBMs) of health-related quality of life [11].

However, many clinical trials do not include a PBM, limiting the ability to conduct economic evaluations. In such cases, statistical procedures may be used to “map” scores on non-preference based outcome measures, such as the FSS, to health state utility values (HSUVs) derived from PBMs. “Mapping” involves regression analysis, using a dataset containing responses to both measures from the same sample, to derive an algorithm that can be used to convert data from non-preference-based measures into HSUVs. Over recent years, the use of mapping has increased considerably [11]. Previous studies have reported on mapping from MS-specific outcome measures including the Multiple Sclerosis Impact Scale and the Multiple Sclerosis Walking Scale-12 [12–14]. However, no approach has been reported that uses fatigue measures to map to HSUVs in the context of MS.

## Methods

This paper uses statistical techniques to map from the FSS (the “source measure”) to HSUVs derived from three preference-based measures: the EQ-5D, SF-6D and MSIS-8D (the “target measures”). The aim is to derive algorithms to convert FSS scores into HSUVs for use in assessing the cost-effectiveness of treatments for fatigue in people with MS. The statistical approach presented here is based on good practice methodology, and is consistent with the recommendations regarding mapping methods from NICE in the UK [15] and the international ISPOR Good Practices for Outcomes Research Task Force [16].

### Measures

*The Fatigue Severity Scale (FSS)* has acceptable reliability, internal consistency, sensitivity and responsiveness for people with MS [3, 17–21]. It comprises nine statements, describing the severity and impact of fatigue, with a scale of possible responses ranging from 1 (“strongly disagree”) to 7 (“strongly agree”). FSS total scores are usually reported as the mean score over the nine items; a higher score indicates greater severity.

*The EuroQoL EQ-5D-3L* has five dimensions (mobility, self-care, usual activities, pain/discomfort, anxiety/depression) with three response levels per dimension - no problems, some problems or extreme problems/confined to bed. HSUVs were derived from the preferences of a representative sample of the UK general population, using a variant of the time trade-off (TTO) technique, and range from − 0.594 to 1.000 [22]. The EQ-5D is widely used in economic evaluations, particularly in the UK, where NICE recommend it as the preferred measure of health outcomes for cost effectiveness analyses [8].

*The Short-Form 6D (SF-6D)* enables HSUVs to be estimated from a popular non-preference based measure of health-related quality of life (HRQoL), the Short-Form 36 (SF-36). It consists of six dimensions (physical functioning, role limitations, social functioning, pain, mental health, vitality) with between four and six response levels. Preferences were elicited from a representative sample of the UK general population using the standard gamble technique and values range from 0.301 to 1.000 [23]. The dataset used for analysis includes responses to Version 1 of the SF-36 from earlier waves of data collection, before this was replaced by SF-36 Version 2, which was developed to address concerns about the structure and wording of some items [24]. Given that the component items of the SF-6D classification system differ between the two versions, we only included responses to Version 2 of the SF-36 in this analysis, in order to ensure consistency.

*The Multiple Sclerosis Impact Scale 8D (MSIS-8D)* enables HSUVs to be estimated from responses to a MS-specific outcome measure, the Multiple Sclerosis Impact Scale (MSIS-29). It includes eight dimensions (physical function, social and leisure activities, mobility, daily activities, mental fatigue, emotional well-being, cognition, depression) with four response levels each [25]. HSUVs were derived from a TTO survey with a sample of the UK general population. Values range from 0.079 to 0.882. It was not assumed that the best health state described by the MSIS-8D classification system (ie “no problems” on all dimensions) was equivalent to perfect health, therefore the value of this health state was not constrained to 1 [26]. The MSIS-8D was derived from Version 2 of the MSIS-29 [21], which has four response levels per item, rather than Version 1 of the MSIS-29, which has five response levels [27]. Therefore, although earlier waves of data collection used Version 1 of the MSIS-29, only responses to Version 2 were included in this analysis.

### Dataset

The South West Impact of Multiple Sclerosis (SWIMS) project is a longitudinal cohort study of people with MS aged 18 or over, living in Devon and Cornwall. Respondents complete six-monthly questionnaires, including several patient-reported outcome measures alongside clinical and demographic characteristics. The study was approved in the UK by the Cornwall and Plymouth and South Devon Research Ethics Committees, and written informed consent is obtained from all participants.

This analysis used SWIMS data received between August 2004 and October 2012. Only data collected at baseline were included, as this is the only point at which the FSS, EQ-5D, SF-36 and MSIS-29 are completed simultaneously. A random sample of 75% of the baseline data were used as the estimation dataset (*n* = 1056), with the remaining 25% constituting the validation dataset (*n* = 352) [11, 28]. As Table 1 shows, there were no significant differences (*p* < 0.05) between the datasets in terms of mean FSS total scores, mean HSUVs, or recorded demographic or clinical characteristics. The mapping algorithms were derived using data from respondents who provided answers to all questions required to produce both a FSS total score and a HSUV from the target PBM: 1023 respondents for the EQ-5D, 607 for the SF-6D and 650 for the MSIS-8D (response numbers are lower for the SF-6D and the MSIS-8D as only version 2 of these questionnaires were included). All statistical analysis was undertaken in Stata 14.

**Table 1:** Summary of respondent characteristics, comparison of estimation and validation datasets

	All baseline data			Estimation dataset			Validation dataset			Difference^a^	
	Mean	SD	Observations	Mean	SD	Observations	Mean	SD	Observations	t statistic	*p* value
Measure											
FSS	43.73	15.10	1054	43.44	15.16	787	44.60	14.91	267	−1.085	0.278
EQ-5D	0.596	0.295	1346	0.600	0.291	1005	0.584	0.309	341	0.831	0.406
SF-6D	0.646	0.130	632	0.650	0.135	473	0.636	0.113	159	1.141	0.254
MSIS-8D	0.646	0.185	690	0.647	0.190	523	0.641	0.172	167	0.412	0.681
Characteristic											
Age	50.67	11.68	1400	50.74	11.73	1048	50.45	11.54	352	0.402	0.688
Duration (years)	9.62	10.01	1347	9.61	10.00	1009	9.68	10.09	338	−0.113	0.910
EDSS score	4.30	2.31	289	4.32	2.34	218	4.22	2.24	71	0.324	0.746
	Percentage		Observations	Percentage		Observations	Percentage		Observations	chi^2^ statistic	p value
Gender											
Female	73.86%		1040	74.62%		788	71.59%		252	1.256	0.262
Male	26.14%		368	25.38%		268	28.41%		100		
MS type											
Relapsing remitting	41.97%		572	42.66%		439	39.82%		133	7.572	0.109
Primary progressive	19.37%		264	18.56%		191	21.86%		73		
Secondary progressive	16.95%		231	17.69%		182	14.67%		49		
Benign	3.3%		45	3.69%		38	2.10%		7		
DK or combination	18.42%		251	17.40%		179	21.56%		72		
Missing			45			27			18		
Recent relapse^b^											
Yes	53.55%		732	53.42%		546	53.91%		186	0.025	0.988
No	33.28%		455	33.37%		341	33.04%		114		
Don’t know	13.17%		180	13.21%		135	13.04%		45		
Missing			41			34			7		

*SD* standard deviation, *FSS* Fatigue Severity Scale, *EQ-5D* EuroQoL EQ-5D-3L, *SF-6D* Short-Form 6D, *MSIS-8D* Multiple Sclerosis Impact Scale – Eight Dimensions, *EDSS* Expanded Disability Status Scale, *DK* don’t know

^a^Difference between estimation and validation datasets

^b^Relapse in the 12 months prior to completing the baseline questionnaire

nb response numbers are lower for the SF-6D and the MSIS-8D as only version 2 of these questionnaires were included

### Preliminary assessment of measures

Two key conditions must be met for mapping: there should be conceptual overlap between the source and target measures, and the target measure should demonstrate discriminative validity with respect to the severity of the condition captured by the source measure [11, 29]. To assess conceptual overlap, the FSS items and the dimensions of the PBMs were allocated to a multi-dimensional conceptual framework, which was developed for this study in order to provide a structure for comparing the content of the measures. The measurement concept underpinning the three PBMs is (HRQoL) [22, 23, 25]. Therefore, the conceptual framework was structured around the commonly agreed key dimensions of HRQL, which comprise physical and mental domains alongside a third domain relating to social and role function and participation [30–32]. The framework was constructed based on a systematic literature review of qualitative research into the impact of fatigue on people with MS (details of this review are included as Additional file 1: A).

Pearson correlation coefficients were assessed between the total FSS score and HSUVs from each of the PBMs, while Spearman correlation coefficients were assessed between FSS total scores and individual dimension scores for each PBM, and between HSUVs and individual FSS item scores. Assuming that these instruments measure distinct but related concepts, we expected to find relationships of moderate strength, ie correlation coefficients between 0.3 and 0.6 [33]. To assess the discriminative validity of the PBMs, respondents were categorised into fatigue severity groups: “mild/ no fatigue” (FSS total ≤ 35), “moderate fatigue” (36 ≤ FSS total ≤ 52) and “severe fatigue” (FSS total ≥ 53). The definition of “mild/ no fatigue” was based on the published cut-off point for the FSS [17]. The ability of the PBMs to differentiate between the three groups was investigated using ANOVA and standardised effect sizes. Effect sizes can be assessed as small (0.20–0.49), moderate (0.50–0.79) or large (0.80 or over) [34].

### Development of mapping algorithms

#### Exploration of model specifications

The relationships between the source and target measures were examined using statistical conventions reported in the mapping literature [29, 35]. The distribution of scores on each of the measures was explored by the production of histograms and, the relationship between each of the PBMS and the FSS total score was investigated by production of scatterplots. Five regression models were estimated for each PBM. HSUVs were regressed on the:

- Total FSS score (Model A);
- Total FSS score and total FSS score squared (Model B);
- Total FSS score, age and gender (Model C);
- FSS item scores (Model D);
- FSS item scores, age and gender (Model E).

The majority of mapping studies estimate algorithms using ordinary least squares (OLS) models [35]. However, OLS models can predict values outside the possible range for a PBM, and can lack predictive accuracy for extreme HSUVs. To address this, Tobit models were also considered, specifying an upper limit of 1 [29]. OLS and Tobit models rely on an assumption of no heteroscedasticity. Where this assumption was violated according to White’s test for heteroscedasticity, the ‘vce(robust)’ option was used in conjunction with the ‘regress’ command for the OLS analyses, and Censored Least Adjusted Deviation (CLAD) estimation methods [36] were used instead of Tobit models, employing the ‘clad’ command with a specified upper limit of 1.

Predictive ability was assessed using the following estimation errors: mean absolute error (MAE), root mean squared error (RMSE) and the proportions of estimates that fell within 0.05, 0.10 and 0.25 of the observed HSUV. MAE was selected as the primary criterion for selection of the preferred models [11]. However, if coefficients had unexpected signs these models were not selected. In instances where model MAEs were the same, the model with the best profile of estimates falling within 0.05, 0.10 and 0.25 of the observed HSUV was selected.

Two researchers decided independently which models to would take forward for validation. Where discrepancies arose, these were resolved through discussion until consensus was reached. Demographic variables may not be included in the datasets from which HSUVs are to be estimated. Therefore, where the best performing models included demographic variables, the best performing model without demographic variables was also selected.

#### Validation and model selection

Estimation errors were assessed according to the severity of the health state. The selected models were applied to the validation dataset and their performance was assessed using the criteria outlined above.

## Results

### Preliminary assessment of measures

**Fig. 1:**
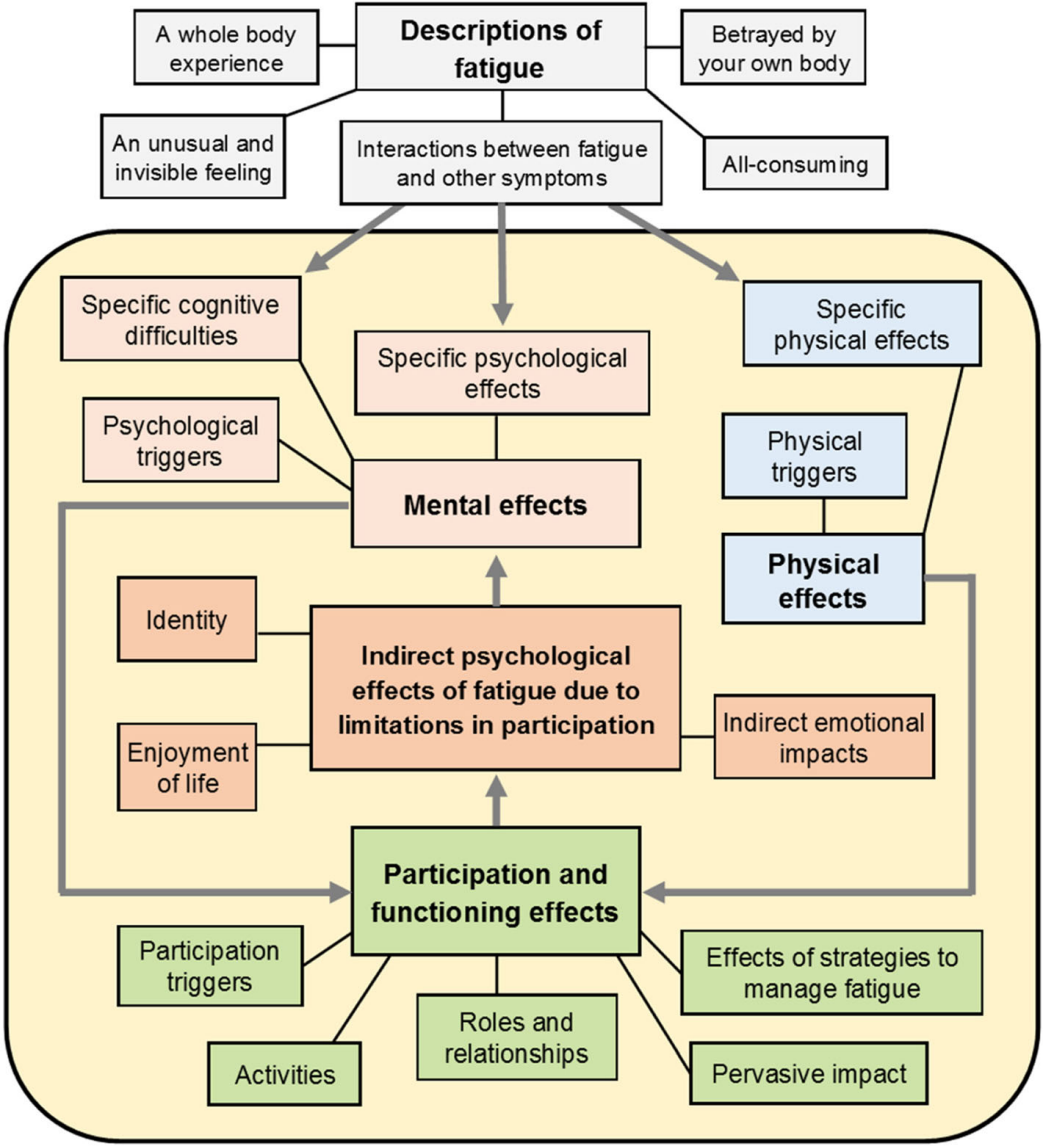
Conceptual framework

The conceptual framework that was developed to assess conceptual overlap between the measures is illustrated in Fig. 1. Most of the themes that had been identified in the original qualitative research studies fitted into the three domains of HRQoL that were defined a priori. There were two notable exceptions. Several of the themes described the experience of fatigue itself, rather than its effect on HRQoL. This experience was clearly of great importance to the people with MS who contributed to the original research, and underpinned the ways in which fatigue impacts upon HRQoL. Therefore, an additional domain was added: “Descriptions of fatigue”. In terms of the links between themes, a clear relationship emerged between “functioning and participation” and “psychological well-being”. People with MS specifically identified negative effects on their psychological well-being that were caused by the impact of their fatigue on their functioning and participation. These stood alongside, but distinct from, the direct impact of fatigue on psychological well-being. Therefore, this became a domain in its own right.

In terms of conceptual overlap, the FSS and all PBMs cover the three primary domains of the conceptual framework (Physical, Mental and Participation Effects) (Table 2). Coverage of Participation Effects is strong across all four measures. The FSS, SF-6D and MSIS-8D capture a wide range of Physical Effects, whereas the EQ-5D includes only specific dimensions for pain/discomfort and mobility. In terms of Mental Effects, the FSS includes one item relating to motivation, while the PBMs describe other specific symptoms eg depression or anxiety. Only the MSIS-8D includes cognitive effects. The MSIS-8D and SF-6D include dimensions relating specifically to fatigue or vitality.

**Table 2:** Comparison of measures against conceptual framework

Conceptual framework	Fatigue severity scale	EQ-5D	SF-6D	MSIS-8D
Descriptions of fatigue				
General fatigue or vitality	3. Easily fatigued 5. Causes frequent problems 8. Among most disabling symptoms	–	6. Vitality	–
Physical effects				
General	4. Interferes with physical functioning 6. Prevents sustained physical functioning	–	1. Physical functioning	1. Physically demanding tasks
Triggers-				
Specific physical effects	2. Exercise brings on fatigue	4. Pain/Discomfort 1. Mobility	4. Pain	3. Being stuck at home
Mental effects				
General	–	–	–	5. Feeling mentally fatigued
Specific psychological effects	1. Motivation is lower	5. Anxiety/Depression	5. Mental health	6. Irritable, impatient, short-tempered 8. Feeling depressed
Specific cognitive effects	–	–	–	7. Problems concentrating
Indirect effects	–	–	–	–
Participation effects				
General	7. Interferes with duties & responsibilities 9. Interferes with work, family, social life		–	3. Being stuck at home
Effects on specific activities		2. Self-Care 3. Usual Activities	1. Physical functioning 2. Role limitations 3. Social functioning 4. Pain	2. Social and leisure activities 4. Work or other daily activities

*EQ-5D* EuroQoL EQ-5D-3L; *SF-6D* Short-Form 6D, *MSIS-8D* Multiple Sclerosis Impact Scale – Eight Dimensions

Explanation for allocation of particular items:

• SF-6D Physical functioning: included under both “Physical effects” and “Functioning/ participation” because level descriptions include “moderate/ vigorous activities” and “bathing and dressing”

• SF-6D Pain: included under both “Physical effects” and “Functioning/ participation” because level descriptions include “pain that interferes with your normal work”

• SF-6D Mental health: included under “Specific psychological effects” because level descriptions refer to feeling “tense or downhearted and low”

• SF-6D Role limitations: included under “Functioning/ participation – activities” because level descriptions refer to “work or other regular daily activities”.

• MSIS-8D Being stuck at home: included under “Specific physical effects” because the MSIS-8D uses this question as a proxy for mobility, however we have also included it here under “Functioning/ participation”

Significant (*p* < 0.0001) moderate correlations were evident between the FSS total score and HSUVs derived from the EQ-5D (*r* = − 0.455) and the MSIS-8D (− 0.590). There was a large significant correlation (*p* < 0.0001) between the FSS total score and HSUVs derived from the SF-6D (− 0.647). The FSS total score was significantly correlated with all individual dimensions of the PBMs, and HSUVs derived from each of the PBMs were significantly correlated with all individual items of the FSS (*p* < 0.0001). Most correlations were moderate, as anticipated, and all had the expected negative sign, ie higher FSS scores are related to lower HSUVs (Table 3).

**Table 3:** Correlations between Fatigue Severity Scale and preference-based measures

FSS total score and PBM dimensions	rho	Observations
EQ-5D versus FSS total score		
Mobility	0.423	1035
Self-care	0.385	1048
Usual activities	0.524	1051
Pain/Discomfort	0.361	1047
Anxiety/Depression	0.292	1049
SF-6D versus FSS total score		
Physical functioning	0.547	649
Role limitations	0.424	645
Social functioning	0.530	644
Pain	0.429	642
Mental health	0.324	648
Vitality	0.615	654
MSIS-8D versus FSS total score		
Physically demanding tasks	0.585	656
Social and leisure activities	0.560	652
Mobility (being stuck at home)	0.489	656
Work or other daily activities	0.558	655
Feeling mentally fatigued	0.582	656
Feeling irritable, impatient or short-tempered	0.377	654
Problems concentrating	0.450	654
Feeling depressed	0.320	653
EQ-5D versus FSS item		
1 My motivation is lower	−0.285	1040
2 Exercise brings on my fatigue	− 0.382	1038
3 I am easily fatigued	− 0.464	1040
4 Interferes with physical functioning	−0.471	1033
5 Causes frequent problems for me	−0.498	1039
6 Prevents sustained physical functioning	−0.527	1040
7 Interferes with duties and responsibilities	−0.536	1038
8 Among my most disabling symptoms	−0.336	1035
9 Interferes with work, family or social life	−0.482	1039
SF-6D versus FSS item		
1 My motivation is lower	−0.400	614
2 Exercise brings on my fatigue	− 0.409	614
3 I am easily fatigued	−0.545	614
4 Interferes with physical functioning	−0.541	612
5 Causes frequent problems for me	−0.585	614
6 Prevents sustained physical functioning	−0.575	614
7 Interferes with duties and responsibilities	−0.623	613
8 Among my most disabling symptoms	−0.455	610
9 Interferes with work, family or social life	−0.603	614
MSIS-8D versus FSS item		
1 My motivation is lower	−0.387	659
2 Exercise brings on my fatigue	−0.423	659
3 I am easily fatigued	−0.560	659
4 Interferes with physical functioning	−0.554	656
5 Causes frequent problems for me	−0.615	659
6 Prevents sustained physical functioning	−0.606	660
7 Interferes with duties and responsibilities	−0.637	660
8 Among my most disabling symptoms	−0.428	656
9 Interferes with work, family or social life	−0.596	659

All coefficients significant at *p* < 0.0001

*PBM* preference-based measure, *FSS* Fatigue Severity Scale, *EQ-5D* EuroQoL EQ-5D-3L, *SF-6D* Short-Form 6D, *MSIS-8D* Multiple Sclerosis Impact Scale – Eight Dimensions

28.4% of respondents with a valid FSS total score were in the “mild/ no fatigue” category, 36.6% were in the “moderate fatigue” category and 35.0% were in the “severe fatigue” category. All PBMs discriminated significantly between fatigue severity groups (*p* < 0.0001). The SF-6D performed particularly well, with large standardised effect sizes (≥0.80). Overall, standardised effect sizes were higher for the MSIS-8D than for the EQ-5D (Table 4).

**Table 4:** Discriminative validity

	Mean	SD	Obs	SES
EQ-5D vs FSS groups				
Mild/no fatigue	0.775	0.218	297	0.615
Moderate fatigue	0.641	0.233	369	0.803
Severe fatigue	0.454	0.3	357	
FFS total	0.614	0.285	1023	
F-statistic	131.84	Prob <	0.0001	
Bartlett’s chi2	40.065	Prob <	0.0001	
SF-6D vs FSS groups				
Mild/no fatigue	0.747	0.124	189	0.871
Moderate fatigue	0.639	0.099	225	0.879
Severe fatigue	0.552	0.083	193	
FFS total	0.645	0.129	607	
F-statistic	172.46	Prob <	0.0001	
Bartlett’s chi2	30.047	Prob <	0.0001	
MSIS-8D vs FSS groups				
Mild/no fatigue	0.764	0.115	202	0.739
Moderate fatigue	0.679	0.134	240	1.381
Severe fatigue	0.494	0.186	208	
FFS total	0.646	0.184	650	
F-statistic	180.71	Prob <	0.0001	
Bartlett chi2	51.434	Prob <	0.0001	

*SD* standard deviation, *obs* observations, *SES* standardised effect size, *FSS* Fatigue Severity Scale, *EQ-5D* EuroQoL EQ-5D-3L, *SF-6D* Short-Form 6D, *MSIS-8D* Multiple Sclerosis Impact Scale – Eight Dimensions

As a result of the preliminary assessments, it was judged that conceptual overlap and discriminative validity were sufficient to proceed with the estimation of mapping models. Overall, the SF-6D and MSIS-8D provide a better fit with the FSS.

### Results of mapping analysis

#### Exploration of model specifications

In order to allow for heteroscedasticity, skewness and kurtosis identified in the data, we fitted robust OLS models and used a CLAD rather than a Tobit specification. (The distribution of scores on each of the measures, and the relationships between scores on the PBMs and the FSS total score is shown in the Additional file 2 B and Additional file 3: C). Thirty models were considered, with Models A to E estimated for each PBM, using both OLS and CLAD specifications.

There was little difference between the predictive ability of the models based on FSS total scores and individual FSS items. In all models, item FSS-08 had a significant coefficient with an unexpected sign, and a majority of the FSS items (ranging from five to seven of the nine items) were not significant predictors of HSUVs. Furthermore, data on individual FSS items may not be available in all potential applications of the mapping algorithms. Therefore selection was restricted to algorithms based on the FSS total score.

##### EQ-5D

CLAD C had the lowest MAE and the highest proportion of individuals with small prediction errors. We also selected CLAD A, as the model which did not include demographic variables with the lowest MAE.

##### SF-6D

OLS B and CLAD B had coefficients with unexpected signs and were, therefore, not selected. We selected CLAD C as it had the next lowest MAE, and OLS A and CLAD A, as they did not include demographic variables.

##### MSIS-8D

CLAD B and OLS B had the lowest MAEs, however these had unexpected signs for FSS total, and so were not selected. The model with the next lowest MAE and highest proportion of individuals with small predictions errors was CLAD C. As this model included demographic variables, we also selected the model with the next lowest MAE (0.117), CLAD A.

Details of the selected models are presented in Table 5. All model results are provided in Additional file 4: D.

**Table 5:** Models mapping from FSS total to PBMs using estimation dataset

	EQ-5D				SF-6D						MSIS-8D			
	CLAD A		CLAD C		OLS A		CLAD A		CLAD C		CLAD A		CLAD C	
	Coeff	SE	Coeff	SE	Coeff	SE	Coeff	SE	Coeff	SE	Coeff	SE	Coeff	SE
FSS total	− 0.006*	0.0006	− 0.006*	0.0006	− 0.006*	0.0003	−0.006*	0.0004	−0.006*	0.0004	−0.007*	0.0007	−0.008*	0.0008
Age			−0.003*	0.0007					−0.0005	0.0005			−0.001	0.0008
Female			0.012	0.0133					−0.012	0.0107			−0.024	0.0233
Constant	0.921	0.0256	1.058	0.0610	0.897	0.0151	0.913	0.0195	0.966	0.0374	0.985	0.0228	1.084	0.0719
Observations	763		755		455		455		452		474		464	
F statistic					357.45									
Prob>F					< 0.0001									
R-squared					0.451									
Pseudo R2	0.107		0.126				0.267		0.274		0.196		0.194	
Coefficients	1		3		1		1		3		1		3	
Significant coefficients	1		2		1		1		1		1		1	
Mean absolute error (MAE)	0.175		0.173		0.078		0.078		0.077		0.117		0.116	
Mean squared error (MSE)	0.066		0.067		0.01		0.01		0.01		0.024		0.023	
Root MSE	0.257		0.258		0.1		0.1		0.1		0.154		0.152	
Normalised root MSE	16.12%		16.19%		14.31%		14.31%		14.31%		19.18%		18.93%	
Individuals with MAE < 0.25	78.37%		79.34%		98.68%		98.68%		98.45%		89.05%		90.41%	
Individuals with MAE < 0.1	47.05%		49.14%		68.13%		69.01%		70.13%		51.93%		51.84%	
Individuals with MAE < 0.05	26.47%		29.14%		41.32%		41.98%		42.48%		28.40%		29.39%	

*Coeff* model coefficient, *SE* standard error, *CLAD* Censored Least Adjusted Deviation model, *EQ-5D* EuroQoL EQ-5D-3L, *SF-6D* Short-Form 6D, *MSIS-8D* Multiple Sclerosis Impact Scale – Eight Dimensions

*p < 0.001

#### Validation and model selection

The validation dataset was used to assess estimation errors for the selected models (Table 6). Table 7 shows MAEs for ‘poor’ and ‘good’ health states by model. The models predicting HSUVs for the EQ-5D and MSIS-8D had larger MAEs for poorer health states, indicating that these models performed less well at estimating scores for those in poorer health states. The opposite was true for the SF-6D models, although the difference in MAEs here was less marked. (Please see Additional file 5: E and Additional file 6: F).

**Table 6:** Models mapping from FSS total to PBMs using validation dataset

	EQ-5D				SF-6D						MSIS-8D			
	CLAD A		CLAD C		OLS A		CLAD A		CLAD C		CLAD A		CLAD C	
	Coeff	SE	Coeff	SE	Coeff	SE	Coeff	SE	Coeff	SE	Coeff	SE	Coeff	SE
FSS total	−0.007*	0.0012	−0.008*	0.0011	−0.004*	0.0005	−0.004*	0.0007	−0.004*	0.0008	−0.006*	0.0010	−0.006*	0.0011
Age			−0.004*	0.0011					0.0004	0.0009			−0.001	0.0020
Female			−0.009	0.0260					0.002	0.0187			0.012	0.0395
Constant	1.001	0.0549	1.233	0.0979	0.81	0.0261	0.793	0.0394	0.827	0.0781	0.939	0.0432	0.974	0.1252
Observations	260		258		152		152		152		157		157	
F statistic					54.71								0.185	
Prob>F					< 0.0001									
R-squared					0.316									
Pseudo R2	0.119		0.141				0.169		0.169		0.180		0.185	
Coefficients	1		3		1		1		3		1		3	
Significant coefficients	1		2		1		1		1		1		1	
Mean absolute error (MAE)	0.183		0.179		0.068		0.068		0.071		0.118		0.114	
Mean squared error (MSE)	0.076		0.071		0.008		0.008		0.009		0.023		0.022	
Root MSE	0.276		0.267		0.09		0.09		0.095		0.151		0.149	
Normalised root MSE	17.31%		16.75%		12.88%		12.88%		13.59%		18.80%		18.56%	
Individuals with MAE < 0.25	78.85%		76.92%		98.68%		98.03%		98.03%		92.36%		91.08%	
Individuals with MAE < 0.1	49.62%		47.31%		76.32%		75.00%		75.66%		50.32%		52.23%	
Individuals with MAE < 0.05	24.62%		25.77%		48.68%		46.05%		46.05%		22.93%		31.21%	

*Coeff* model coefficient, *SE* standard error, *CLAD* Censored Least Adjusted Deviation model, *EQ-5D* EuroQoL EQ-5D-3L; *SF-6D* Short-Form 6D; *MSIS-8D* Multiple Sclerosis Impact Scale – Eight Dimensions

*p < 0.001

**Table 7:** Mean absolute errors by severity group

	CLAD Model A	CLAD Model C	OLS Model A
FSS to EQ-5D			
EQ_5D < =0.65	0.234	0.238	
EQ_5D > 0.65	0.123	0.115	
FSS to SF-6D			
SF_6D < =0.65	0.070	0.070	0.070
SF_6D > 0.65	0.088	0.088	0.088
FSS to MSIS-8D			
MSIS_8D < =0.7	0.154	0.154	
MSIS_8D > 0.7	0.082	0.082	

Cut-off points for EQ-5D, SF-6D and MSIS-8D were chosen to give roughly equally-sized groups

## Discussion

Here we describe and demonstrate a method for converting responses to the FSS, a frequently-used measure of fatigue severity, into HSUVs, which can be used to estimate QALYs for use in cost-effectiveness analyses, and hence to inform decision-making regarding the availability of treatments for MS-related fatigue. According to the Oxford Health Economics Research Centre’s Mapping Database, last updated in April 2019 [37], no previous published studies have attempted mapping from the FSS. In addition, we have found no previous studies which have investigated correlations between the FSS and the SF-6D or the FSS and the MSIS-8D, and just two which have explored the relationship between the FSS and the EQ-5D [38, 39]. Rosa et al. [39] correlated FSS total scores with participants’ scores on the EQ-5D visual analogue scale, rather than with the EQ-5D HSUVs that are relevant for mapping, and Tremmas et al. [38] found no statistically significant correlation between the FSS and EQ-5D scores of people with lung cancer.

The ability of the models selected in the current study to predict SF-6D and MSIS-8D values is in keeping with results reported in other mapping studies [35]. There are currently no guidelines regarding acceptable limits for estimation errors [13], but MAEs ranging from 0.0011 to 0.19 have been previously described [35]. In the current study, the SF-6D MAEs of 0.078 and 0.077 and the MSIS-8D MAEs of 0.117 and 0.116, fall well within this range and, specifically in the context of MS, they are in keeping with the MAE of 0.058 reported by Hawton et al. [12] when the MSIS-29 was mapped to the SF-6D.

Results for the EQ-5D algorithms were less convincing. The prediction errors of 0.175 and 0.173 are towards the higher end of MAEs reported in previous mapping studies [35], and are also high in the context of MS mapping studies. Versteegh et al. [13] mapped from the version 1 of the MSIS-29 to the EQ-5D, with resulting MAEs of 0.13 and 0.16, and Hawton and colleagues [12] mapped from version 2 of the same measures to the EQ-5D with a MAE of 0.147. In addition, when testing the external validity of the Versteegh et al. [13] algorithm, Ernstsson et al. [40] reported a MAE of 0.12.

Information is inevitably lost in the process of mapping, as the resulting algorithm will only reflect the areas of content that overlap between the starting and target measures. This information loss is accentuated when a domain-specific, condition-specific measure, such as the FSS, is mapped to a generic, multi-dimensional measure, such as the EQ-5D. Therefore, greater predictions errors might be anticipated when mapping from such a uni-dimensional scale as the FSS than when mapping from a multi-dimensional scale such as the MSIS-29 [41]. However, this does not appear to hold in the MS mapping literature to date, with Hawton et al. [14] reporting a MAE of 0.148 when they mapped from the MS Walking Scale-12 (a mobility-specific, MS-specific measure) to the EQ-5D, and Sidovar et al. [42] described an error statistic of 0.109 when mapping to/from these same measures.

In the current study, the EQ-5D algorithms were particularly problematic for HSUVs below 0.65. They did not predict any values below 0.54 (assuming an age of 50 years and female gender for CLAD Model C), which is of particular concern for a measure with a minimum value of − 0.594.

On the basis of the statistical assessments reported here, the qualitative assessments of conceptual validity, and setting our findings in the context of other mapping studies in MS and mapping studies more generally, we suggest the use of the following algorithms for mapping from the FSS to HSUVs.

SF-6D estimate = 0.897–0.006*FSS total score

MSIS-8D estimate = 1.084–0.008*FSS total score – 0.001*age – 0.024*gender [0 male, 1 female] or if age and gender are not available:

MSIS-8D estimate = 0.985–0.007*FSS total score

Based on these same assessments, we suggest the EQ-5D algorithms are far less likely to produce accurate or valid estimates of EQ-5D scores.

There are a number of potential limitations of this work. Firstly, the SWIMS data were collected prior to the development and use of the EQ-5D-5L and the mapping algorithms were based on the ‘older’ EQ-5D-3L. It may have been expected that the EQ-5D-5L would supersede the EQ-5D-3L as it was developed with five, rather than the original three, levels in an attempt to improve its responsiveness. However, the English HSUV set for the EQ-5D-5L is not in common use, and if using the EQ-5D-5L descriptive system, the current ‘position statement’ of NICE is to use a cross-walk algorithm to provide HSUVs from the EQ-5D-3L value set. Secondly, the SF-6D value set is based on the use of standard gamble to elicit preferences for health states. This may result in higher HSUVs (than the EQ-5D), as respondents tend to be risk adverse. Thirdly, we did not explore the performance of some of the ‘newer’ mapping model specifications, such as limited dependent variable mixture models or beta-based regression, which may have better accounted for the bi-modal nature of the EQ-5D data. There is some empirical evidence in support of these models, but the ISPOR Task Force report [16] does not advocate any specific regression approach for mapping, recognising that the performance of different methods will vary dependent on a number of factors including the nature of the starting/target measures, the disease, and the patient population. The report suggests it is wise to use a model type for which there is existing evidence of good performance. In the context of MS, mapping algorithms which have used the same regression approaches that we have used here have been reported with MAEs of 0.058 [12], 0.13 and 0.16 [13], 0.147 [12], 0.12 [40], 0.148 [14] and 0.109 [42]. Brazier et al.’s [35] systematic review of mapping studies reported MAEs of 0.0011 to 0.19. Therefore, the regression approaches in the current paper have a track record of use and acceptability in the context of MS. The MAEs reported here for the SF-6D and MSIS-8D are in keeping with those reported in these other mapping studies. The poor performance of the EQ-5D algorithms is likely to be a function of the limited conceptual overlap between the EQ-5D and the FSS. The limited shared conceptual content of these measures will not be altered by using a different form of regression analysis. Thirdly, algorithms to predict HSUVs from individual FSS items, rather than the total score, were not generated by this study. This was, in part, due to an anomaly affecting item FSS-08 (Fatigue is among the most disabling of my symptoms). While the item correlated negatively (as expected) with HSUVs when considered in isolation, it had a positive coefficient when included as an independent variable in regression analysis. Further research would be required to understand the mechanisms behind this; in the meantime, it is not possible to determine whether this item is suitable for inclusion in a mapping algorithm.

A particular strength of this study is the nature of the SWIMS dataset. It has provided comprehensive data on which to base the estimation and validation of these mapping algorithms. Importantly, the cohort is comparable with other UK-based samples of people with MS in terms of age, gender, relapse rates and duration of illness [8, 43–47], meaning the algorithms should apply generally to people with MS, rather than just to specific sub-groups. In addition, the work undertaken to explore the content overlap between the measures provided a form of ‘triangulation’ in assessing the appropriateness of the mapping algorithms. Drawing on good quality qualitative research findings regarding the impacts of fatigue on HRQoL and developing a conceptual framework, provided unique insights into why the measures did and did not map well.

It is acknowledged that mapping methods are a second-best option to directly collected HSUVs for estimating QALYs [29, 41, 48]. Use of mapping increases the uncertainty and error around estimates of HSUVs [29], and is particularly problematic when there is little content overlap or relationship between the measures being mapped to and from [41]. However, when PBM data are not collected directly in a trial, empirically-evidenced mapping algorithms may be used. With the exception of the EQ-5D, the algorithms reported here can be used to support improvements in decision-making where primary PBM data are unavailable.

## Conclusions

We present statistical algorithms that allow data from the FSS, a fatigue-specific patient-reported outcome measure, to be used in the estimation of QALYs, which are a suitable and policy-relevant measure for use in cost-effectiveness analyses. This will enable the results of studies using the FSS to inform decision-making in a health technology assessment context.

## Additional files

Additional file 1: Development of a conceptual framework describing the impact of fatigue on people with MS: a systematic review of the literature. (DOCX 163 kb)

Additional file 2: Histograms of source and target measures. (DOCX 119 kb)

Additional file 3: Scatterplots of FSS and PBM scores. (DOCX 204 kb)

Additional file 4: All model results. (XLSX 52 kb)

Additional file 5: Scatterplots of observed vs predicted HSUVs. (DOCX 320 kb)

Additional file 6: Observed versus predicted HSUVs by severity. (XLSX 359 kb)

## Data Availability

The data that support the findings of this study are available from SWIMS Data-Sharing Committee.

## Abbreviations

CLAD: Censored least absolute deviation
EQ-5D: EuroQoL EQ-5D-3L
FSS: Fatigue Severity Scale
HSUV: health state utility value
MS: multiple Sclerosis
MSIS-8D: Multiple Sclerosis Impact Scale-8D
NICE: National Institute for Health and Care Excellence
OLS: Ordinary least squares
PBM: preference-based measure
QALY: quality-adjusted life-year
SF-6D: Short-Form 6D
SWIMS: South West Impact of MS study
TTO: Time trade-off
