# Supplementary material for: Using the Fatigue Severity Scale to inform healthcare decision-making in multiple sclerosis: mapping to three quality-adjusted life-year measures (EQ-5D-3L, SF-6D, MSIS-8D)
Source: Health Qual Life Outcomes. 2019 Aug 5;17:136. doi: 10.1186/s12955-019-1205-y (PMC6683407; doi:10.1186/s12955-019-1205-y)
Supplement: Supplementary file 1 — Development of a conceptual framework describing the impact of fatigue on people with MS: a systematic review of the literature. (DOCX 163 kb) [file 12955_2019_1205_MOESM1_ESM.docx]

**Additional file 1**

**Development of a conceptual framework describing the impact of fatigue on people with MS: a systematic review of the literature**

Aim: to identify the main impacts of fatigue on the quality of life of people with MS, from the perspective of people with MS.

Objective: to review the qualitative literature on the impact of fatigue on the lived experiences of people with MS

**Methods**

**Literature search methods**

A search design was developed based on three key components of the objective of the literature review: multiple sclerosis, fatigue and qualitative methods.

MS search terms were based on those used for routine searches undertaken by the Cochrane Collaboration’s “Multiple sclerosis and rare diseases of the central nervous system” group [Cochrane 2017].

Fatigue search terms were based on those used in a review of interventions for fatigue in Parkinson’s disease undertaken by the Cochrane Movement Disorders Group [Elbers et al, 2014].

Qualitative search terms were based on those developed for the purposes of a study that investigated how to find qualitative research in the context of the medical literature [Shaw et al, 2004].

Search terms within each component were combined using the Bayesian operator “or”. The components were combined using the “and” operator.

Inclusion criteria

- Original research using a qualitative methodology
- Participants are people with MS, or include people with MS alongside people with other conditions, where the results for people with MS are separately identifiable
- Papers with a stated aim of investigating the impact of fatigue on one or more aspects of (health-related) quality of life, well-being, functioning or participation
- English language

Exclusion criteria

- Review papers
- Papers that explore one or more aspects of (health-related) quality of life, well-being, functioning or participation in MS, without an *a priori* focus on fatigue.
- Papers that focused on fatigue, but did not report on the impact of fatigue on (health-related) quality of life, well-being, functioning or participation.
- Papers that did not present separately identifiable results for people with MS.

**Analysis methods**

The purpose of this review is to provide the background for mapping between the FSS and the EQ-5D, SF-6D and MSIS-8D. The measurement concept underpinning the three selected PBMs is health-related quality of life. Therefore, the results of the literature review were analysed using a conceptual framework based on the key dimensions of health-related quality of life.

There is no firm agreement on which dimensions comprise HRQoL, however there is a consensus that, at a minimum, physical and psychological domains should be included [Riazi, 2006]. More recently, a third dimension, relating to social and role function and participation (ie one’s ability to perform ‘normal’ or expected activities and roles), has been added [Ware, 2003]. Therefore, the conceptual framework consisted of three broad domains: physical functioning, psychological and cognitive functioning, and social functioning and participation.

The themes that were identified by the original analysis were extracted from the results sections of the papers and pasted into tables, including the name of the theme and a description of its contents. All identified themes were then allocated to the three domains of HRQoL. Any themes that were repeated across more than one study were combined and any links between themes were noted. Themes that did not fit into the HRQoL domains were collated separately.

In the next stage, sub- domains were developed by grouping together themes that described similar concepts within each domains of HRQoL. The themes that did not fit into the HRQoL domains and the links between themes were explored to determine whether amendments needed to be made to the three-dimensional structure of the conceptual framework. This was then used to produce a conceptual framework of how fatigue affects the HRQoL of people with MS, for use in assessing the content validity of the source and target measures.

**Results**

**Literature search results**

The literature search returned 1124 results. Based on the titles and abstracts of these, 1062 were excluded from further consideration. Of the remaining 62 studies, 11 were conference abstracts for which the full text was not available. The full text of the remaining 52 papers was obtained, and these were assessed against the inclusion and exclusion criteria. Seventeen papers were excluded because they focussed on aspects of fatigue other than its impact (n=7), they did not focus on fatigue (6), they did not use qualitative methods (3) or they were not primarily concerned with MS. Therefore, twelve papers remained for inclusion in the review. This is summarised in Figure A1.


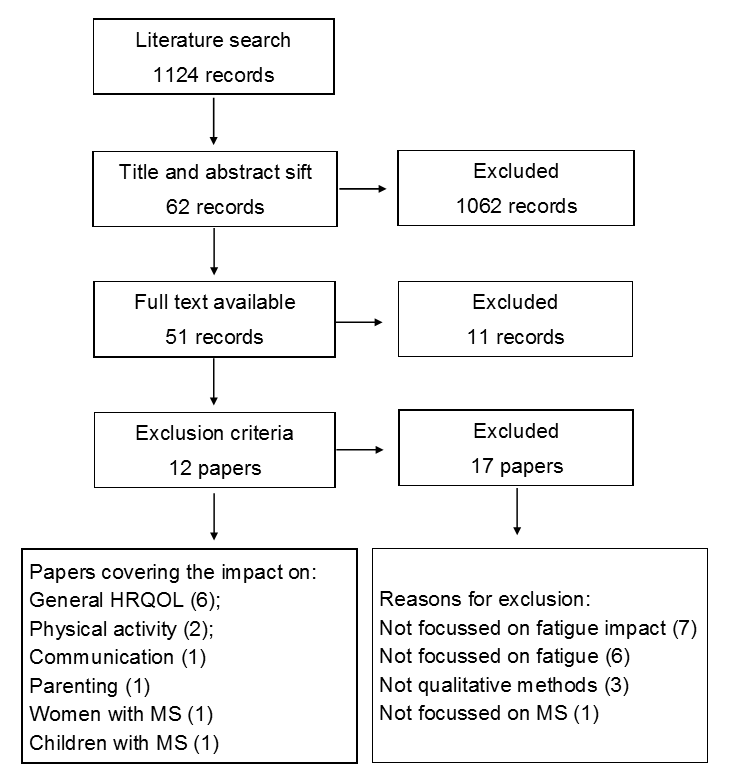


***Figure A1: Literature search results***

**Development of the conceptual framework**

Most of the themes that had been identified in the original qualitative research studies fitted into the three domains of HRQoL that were defined *a priori*. There were two notable exceptions. Several of the themes described the experience of fatigue itself, rather than its effect on HRQoL. This experience was clearly of great important to the people with MS who contributed to the original research, and underpinned the ways in which fatigue impacts upon HRQoL. Therefore, an additional domain was added: “Descriptions of fatigue”. In terms of the links between themes, a clear relationship emerged between “functioning and participation” and “psychological well-being”. People with MS specifically identified negative effects on their psychological well-being that were caused by the impact of their fatigue on their functioning and participation. These stood alongside, but distinct from, the direct impact of fatigue on psychological well-being. Therefore, this became a domain in its own right.

Tables A1 – A4 outline how the themes identified from the literature were mapped to the conceptual framework. The conceptual framework is illustrated in Figure A2.

**Table A1. Descriptions of fatigue**

| **1.1 Fatigue as a whole body experience** | Fatigue was experienced in the muscles, head, and entire body. It affects sensation in the whole body, from the hair to the toes. |
| --- | --- |
|  | Fatigue was perceived in the body – whole of parts of the body were perceived differently than they were before. The body did not feel natural and could not be taken for granted; increased awareness of the body all the time. Strained body with diminished power. |
|  | Two opposite perceptions of the body: (1) heavy & painful (2) numbed, dead, not quite awake, as if parts were missing. |
|  | A feeling of having a heavy body; wanting to let their arms and hands hang down; impossible to raise the arms or hold the body up straight. Muscles feel too weak to support the body |
| **1.2 Betrayed by your own body** | Some felt betrayed when fatigue invaded the body. The body was hard to control and couldn’t be trusted. |
|  | Body will not obey, eg try to lift leg and nothing happens. |
|  | Their own bodies ruled them and they had to adjust themselves. Feeling feeble and unable to manage. |
| **1.3 All-consuming fatigue** | Participants experienced fatigue much of the time, and when they did not, they were thinking about it – always taking it into account. |
|  | An ever-present, ongoing experience, unrelenting and virtually ever present, even after rest or sleep. |
|  | A paralyzing force; although some small reserve of energy is still available, they feel virtually powerless to perform desired activities. |
|  | Undertow Effect: suffocating fatigue characterized by energy impoverishment and absolute powerlessness, relieved only by sleep. |
|  | Energy loss was very unpleasant, perceived as a form of paralyses and as an unstoppable destructive force invading the body, leaving participants unable to manage anything further. |
| **1.4 An unusual and invisible feeling** | A unique and novel sensation, an experience that is different from experiences of being tired when healthy |
|  | Fatigue is invisible and difficult to describe |
| **1.5 Characteristics of the experience of fatigue** | Weariness, sleepiness, tired most days, weak at rest, exhausted after minimal activity |
|  | Sudden, can happen very rapidly, unpredictable, uncontrollable |
|  | Sensation of one’s batteries running out |
|  | Need day rest or sleep |
|  | Unrefreshing or broken nocturnal sleep |
| **1.6 Interactions with other symptoms** | Interaction of fatigue with other symptoms leads to difficulties |
|  | Fatigue that worsens along with other symptoms |
|  | Fatigue can exacerbate other MS symptoms and vice versa |
| Individuals were affected differently by fatigue, and could experience fatigue in one or more different ways during the course of their MS. | |
| It was often hard to differentiate whether a participant was discussing fatigue or MS, as these terms seemed to be used interchangeably | |
| Many participants had experienced more than one state of fatigue during the course of their MS and, on occasion, one state of fatigue could trigger another. | |
| Rather than isolating fatigue, it’s more about the complex and unpredictable relationship between fatigue and other symptoms. | |

**Table A2. Physical effects of fatigue**

| **2.1 Specific physical effects** | Difficulty walking |
| --- | --- |
|  | Falling over |
|  | Weakness/ muscle weakness |
|  | Participants described different states of fatigue. |
|  | Limbs heavy |
|  | Speech problems |
|  | Coordination |
|  | Sensory disturbances/ visual disturbances |
|  | Flickering and swimming or pounding sensation in their eyes, causing dizziness and nausea. |
|  | Pain |
|  | Balance. |
|  | A temporary increase in physical symptoms was associated with increased fatigue. |
| **2.2 Physical triggers** | Physical exertion induces weakness/ worsens fatigue |
|  | Fatigue due to unexpected actions |
|  | The feeling of being fatigued increased because of the extra effort of arranging footsteps when walking. |

**Table A3. Mental effects of fatigue**

| 3.1 Psychological effects of fatigue | Emotional impact of fatigue | |  |
| --- | --- | --- | --- |
|  | A temporary increase in emotional symptoms was associated with increased fatigue. | |  |
|  | Participants described different states of fatigue including feelings of depression. | |  |
|  | Feelings of defeat | |  |
| 3.2 Cognitive effects of fatigue | General | Cognitive impact of fatigue | |
|  |  | Participants described different states of fatigue including mental fogginess. | |
|  |  | Felt that their brain was not totally clear; felt like being struck in the head by a sledgehammer. | |
|  |  | Head experiences: “Brain-cheese,” a “hazy, out-of-body fatigue feeling,” and a “hangover.” | |
|  |  | Links to other symptoms, including cognition. | |
|  |  | A temporary increase in cognitive symptoms was associated with increased fatigue. | |
|  |  | Rather than isolating fatigue, it’s about the complex and unpredictable relationship between fatigue and cognition. | |
|  | Specific | Difficulty concentrating or thinking clearly | |
|  |  | Perception of lower cognitive ability and energy | |
|  |  | Impact on daily life: difficulties in making decisions and plans | |
|  |  | Difficulty solving complex problems | |
|  |  | Difficulty withstanding disturbing sounds | |
|  |  | Difficulties in remembering | |
|  |  | Making mistakes | |
|  |  | Only for brief moments could they feel totally focused. | |
|  |  | Not being able to look forward in time, thinking in the present moment. | |

| **3.3 Psychological triggers of fatigue** | Cognitive | Mental effort worsens fatigue |
| --- | --- | --- |
|  | Emotional | Fatigue due to a change in mood |
|  |  | Stress/anxiety worsens fatigue |
|  |  | Vicious circle: thinking/ worrying about fatigue could cause fatigue, leaving them unable to complete the task |

| **3.4 Indirect psychological effects of fatigue**  **(links to participation and functioning effects)** | Emotional impacts | A feeling of having the will but not the ability: wanted to live life as before and be an active person. |
| --- | --- | --- |
|  |  | Anxiety |
|  |  | Helpless and exposed. |
|  |  | Insecurity |
|  |  | Frustration, stress, sadness. |
|  |  | Dissatisfaction |
|  |  | Lower self-worth, despair, sorrow |
|  |  | Shame; being misunderstood (eg being mistaken as being drunk). |
|  |  | Anger |
|  | Enjoyment of life | Involuntary isolation |
|  |  | Inability to enjoy social activities or hobbies |
|  |  | No fun in life, feeling bored. |
|  |  | Feeling trapped by having to live a very structured life; loss of spontaneity |
|  |  | Prevention of a “normal” life due to fatigue |
|  |  | Forced interruption of activities due to fatigue |
|  |  | Inadequate satisfaction of one’s basic needs |
|  | Identity | Loss of sense of self due to fatigue |
|  |  | Disappointment in a fatigued self |
|  |  | Inability to tend to appearances due to fatigue |
|  |  | Non-achievement of goals due to a gap in the expected and actual behavioral potential |
|  |  | Loss of control, which appeared to threaten the self-integrity of the individual. |
|  |  | Progressive losses including work, youth, driving, strength and energy, relationship roles; feeling “old before my time.”  Losses of driver’s license and employment had emotional effects and challenged men’s self-identities within the family.  Some felt they had progressively lost strength and energy, attributes they linked to “being a man.”  Some described attributes associated with self-identity that either contributed to fatigue or helped them continue their exercise despite fatigue, ie stubbornness, determination. |
|  | Those who were able to engage in valued activities—even if the intensity was less, or if they achieved them through a different route—experienced positive feelings and a sense of control. Those who were unable to make goal adjustments disengaged from valued activities, resulting in negative feelings. | |
|  | Ongoing frustration can lead to depression, particularly “when the frustrated goal is deeply connected to the core of the self”. | |
|  | ‘The importance of having goals that were highly valued and related to activities and work prior to diagnosis allowed the men to feel a sense of achievement and optimism despite their losses.’ | |

**Table 4. Effects of fatigue on participation and functioning**

| **4.1 Pervasive impact** | Influences all activities and responsibilities at work, home, and play. |
| --- | --- |
|  | Restrictions or interruptions to life, including changes in roles within the family, social life and working situation. |
|  | Barriers to participation were not perceived to directly result from any single MS impairment eg fatigue or communication, but from a complex interplay between the impairments experienced by an individual, the coping strategies employed and people’s attitudes. |

| **4.2 Activities** | Put things off, force self to do things |
| --- | --- |
|  | Unable to carry out daily tasks as could before |
|  | Activities of daily living |
|  | Housework |
|  | Giving up work, working fewer hours |
|  | Decreased opportunities for social interaction. |
|  | Social activities/ hobbies |

| **4.3 Effects of strategies to manage fatigue** | Implications of having to plan ahead/ lead structured daily life/ build rest periods into daily routine = less opportunity for spontaneity |
| --- | --- |
|  | Implications of having to reduce overall activity or prioritise certain activities over others = dilemmas over which things don’t get done |
|  | Implications of having to take a planned or necessary cessation of physical activity |
|  | Difficulty of employment due to the measures for treating fatigue, related to an interruption of activities |
|  | May take up formal exercise, or other physical activities, in attempt to enhance resistance to fatigue |
|  | Time-consuming: can’t hurry, need to take time and avoid stressful situations; doing things in advance, calmly and methodically. |

| **4.4 Roles and relationships** | Communication and fatigue | Difficult for others to understand the person’s experiences and needs because fatigue is “invisible” and difficult to describe |
| --- | --- | --- |
|  |  | Fatigue increases the frequency and severity of communication symptoms, language-processing deficits, motor speech symptoms** |
|  |  | Some communication symptoms occur only when experiencing fatigue - language processing difficulties and dysarthria** |
|  |  | The interplay between fatigue and communication led to communication symptoms becoming more apparent to listeners. The resulting communication did not reflect how they would like to represent themselves (eg drunk or lazy rather than able and competent).** |
|  |  | Common to all participants was the enormous effort and pre-planning that remains hidden from communication partners, eg dealing with word finding and memory difficulties, keeping interactions operating as normally as possible on the surface.** |
|  | Handling fatigue in relation to others | Concealment, eg measures to limit activity without letting others know |
|  |  | Measures to arrange an environment by gaining the support of others |
|  | A feeling of being absent | They felt as if they had been split in two parts: one part was participating while the other was just watching.  Feeling both present and absent: seeing everything but feeling as if they weren’t there. Feeling anaesthetized; things just passing by. |
|  |  | Unable to understand things happening around them or to participate in conversations due to lack of concentration. |
|  | Letting people down/ causing problems | Feeling unreliable and could not always keep promises. Leaving everything half-done due to unpredictable fatigue. |
|  |  | Unable to participate in family activities; felt this was difficult for the rest of the family - the whole family was suffering. |
|  |  | Problems in one’s life and friendships due to unpredictable fatigue |
|  |  | Concern of causing friends trouble due to fatigue |
|  | Dependency | Perceptions of dependency - trapped in the sense of needing help from other people – involves feelings of being a burden |

| **4.5 Participation triggers** | Trying to accomplish too much |
| --- | --- |
|  | Family, work or socioeconomic stress |
|  | Continuous nature of burdens and actions |
|  | Work |

**
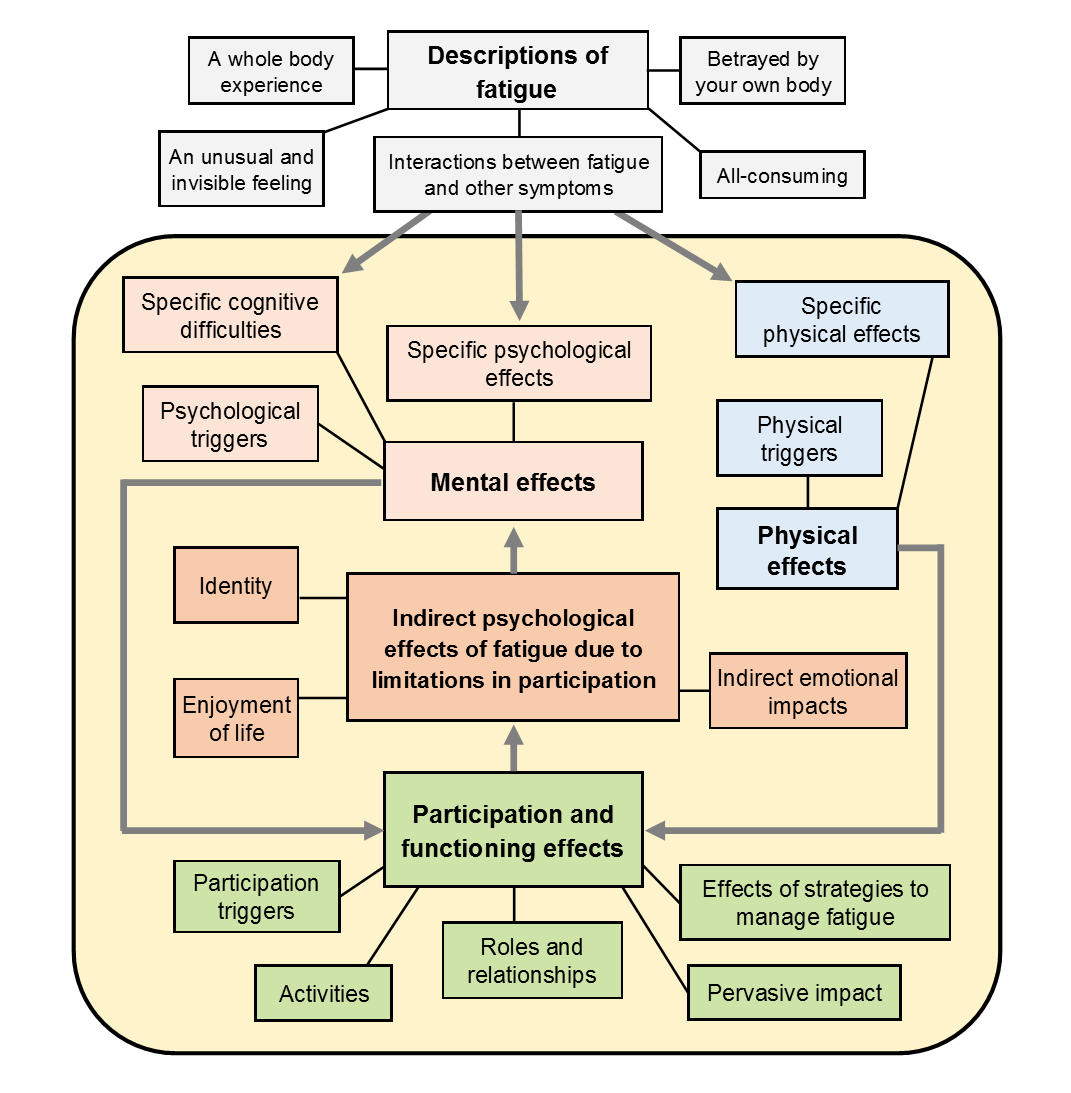
**

**Figure A2: Conceptual framework of the impact of fatigue on people with MS**

**References**

Cochrane 2017: <http://msrdcns.cochrane.org/our-review>, accessed 15/06/2017

Elbers RG, Verhoef J, van Wegen EEH, Berendse HW, Kwakkel G: Interventions for fatigue in Parkinson's disease. 2014: <http://onlinelibrary.wiley.com/doi/10.1002/14651858.CD010925/full> accessed 22/07/2015

European Medicines Agency. Reflection paper on the regulatory guidance for the use of health-related quality of life (HRQL) measures in the evaluation of medicinal products. EMEA/CHMP/EWP/139391/2004. London: European Medicines Agency; 2005.

Riazi A. Patient-reported Outcome Measures in Multiple Sclerosis. The International MS Journal. 2006;13:92–99.

Shaw RL, Booth A, Sutton AJ, Miller T, Smith JA, Young B, Jones DR, Dixon-Woods M. Finding qualitative research: an evaluation of search strategies. BMC Medical Research Methodology 2004, 4:5

Ware J. Conceptualization and measurement of health-related quality of life: comments on an evolving field. Archives of Physical Medicine and Rehabilitation. 2003;84(Suppl 2):S43-S51.
