# Supplementary figures and images for: Using the Fatigue Severity Scale to inform healthcare decision-making in multiple sclerosis: mapping to three quality-adjusted life-year measures (EQ-5D-3L, SF-6D, MSIS-8D)
Source: Health Qual Life Outcomes. 2019 Aug 5;17:136. doi: 10.1186/s12955-019-1205-y (PMC6683407; doi:10.1186/s12955-019-1205-y)

**Additional file 2**

**Histograms of source and target measures**


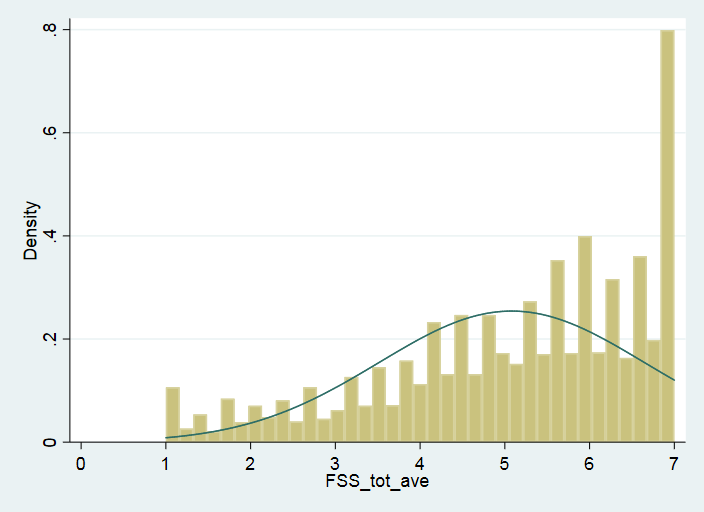

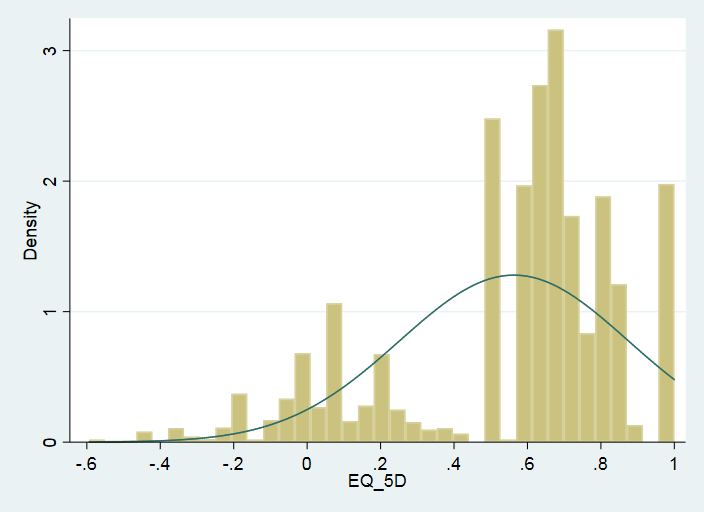


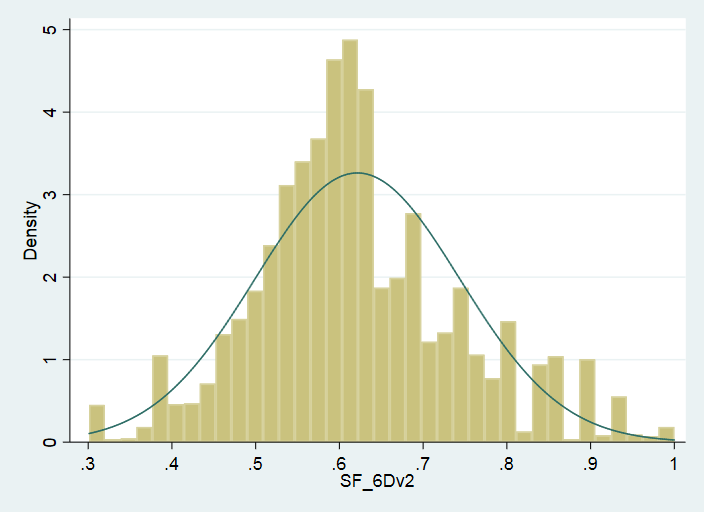

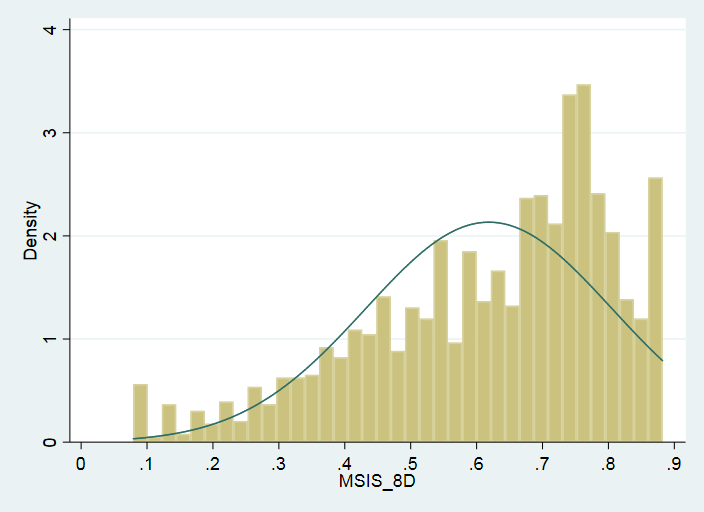

Supplement: Supplementary file 2 — Histograms of source and target measures. (DOCX 119 kb) [file 12955_2019_1205_MOESM2_ESM.docx]

**Additional file 3**

FSS total versus EQ-5D values


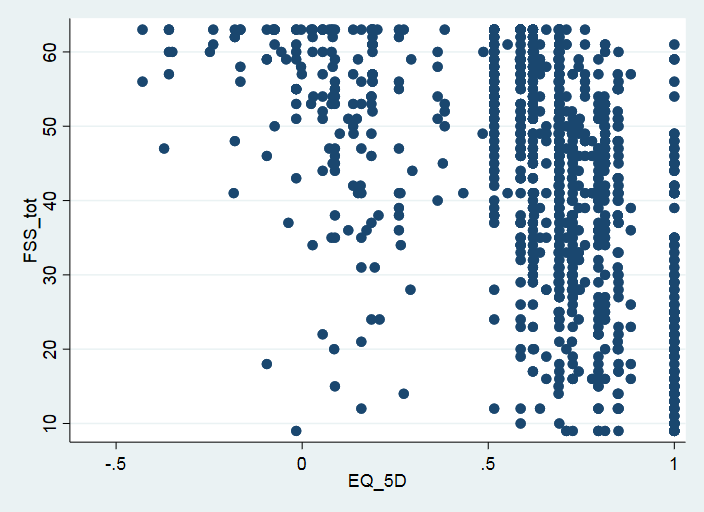


FSS total versus SF-6D values


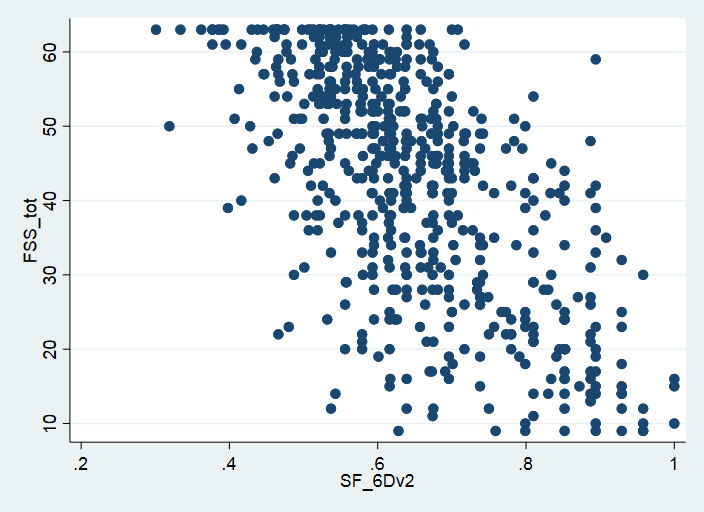


FSS total versus MSIS-8D


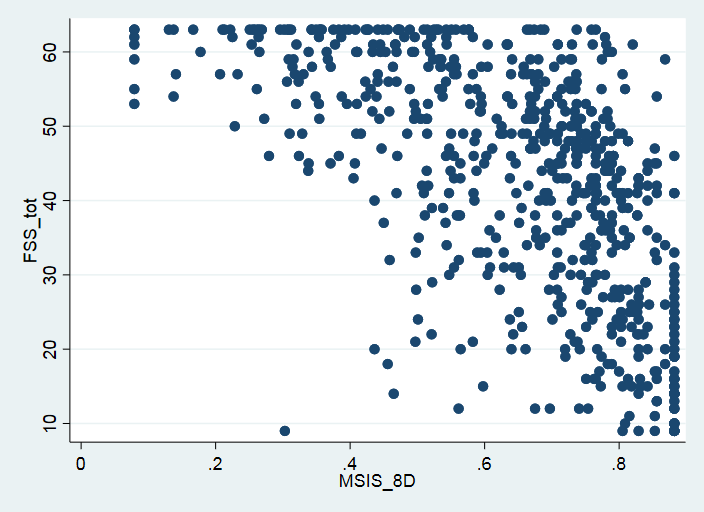

Supplement: Supplementary file 3 — Scatterplots of FSS and PBM scores. (DOCX 204 kb) [file 12955_2019_1205_MOESM3_ESM.docx]
