# Supplementary material for: Using the Fatigue Severity Scale to inform healthcare decision-making in multiple sclerosis: mapping to three quality-adjusted life-year measures (EQ-5D-3L, SF-6D, MSIS-8D)
Source: Health Qual Life Outcomes. 2019 Aug 5;17:136. doi: 10.1186/s12955-019-1205-y (PMC6683407; doi:10.1186/s12955-019-1205-y)
Supplement: Supplementary file 5 — Scatterplots of observed vs predicted HSUVs. (DOCX 320 kb) [file 12955_2019_1205_MOESM5_ESM.docx]

**Additional file 5**

**Scatterplots of observed vs predicted HSVs**


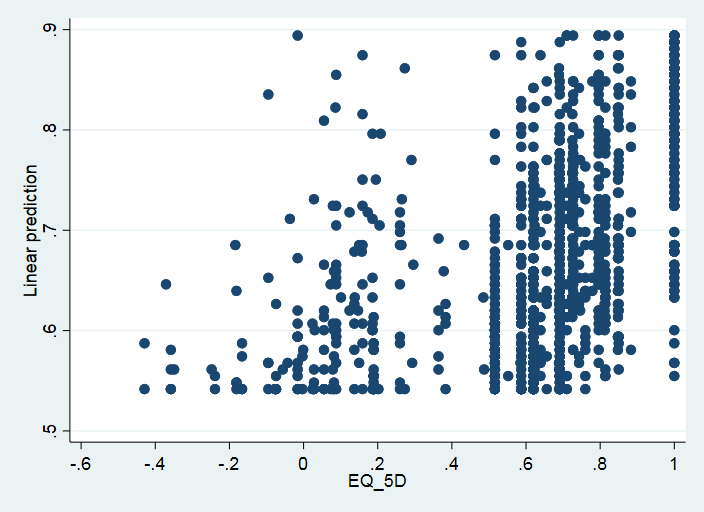

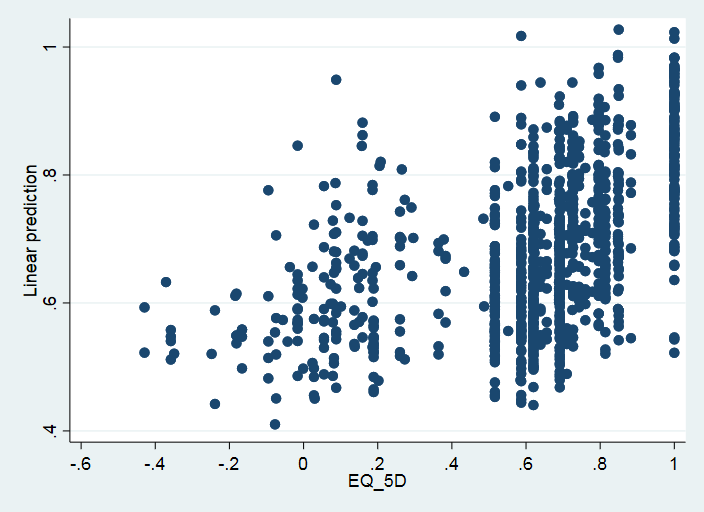


Observed EQ-5D vs EQ-5D estimated using CLAD A Observed EQ-5D vs EQ-5D estimated using CLAD C


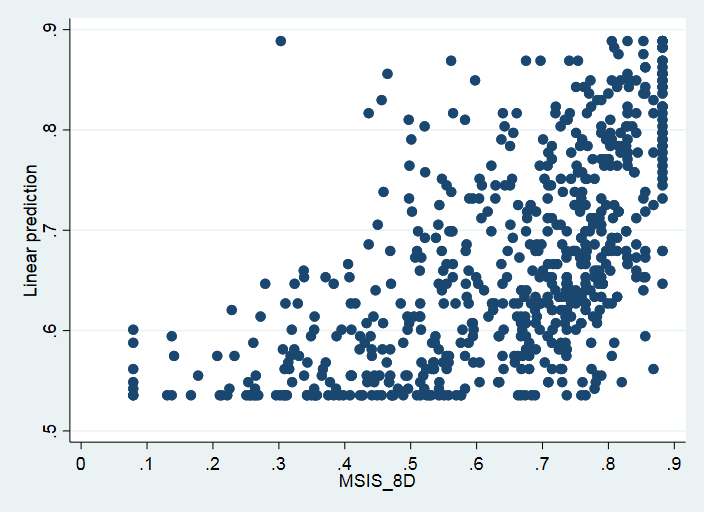

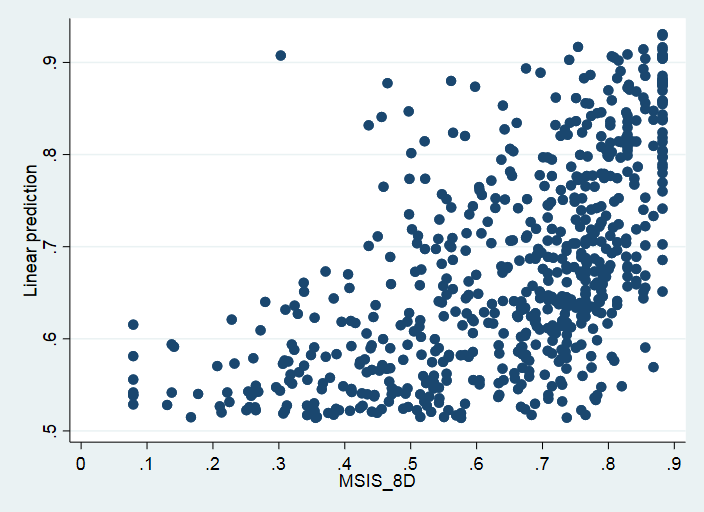


Observed MSIS-8D vs MSIS-8D estimated using CLAD A Observed MSIS-8D vs MSIS-8D estimated using CLAD C


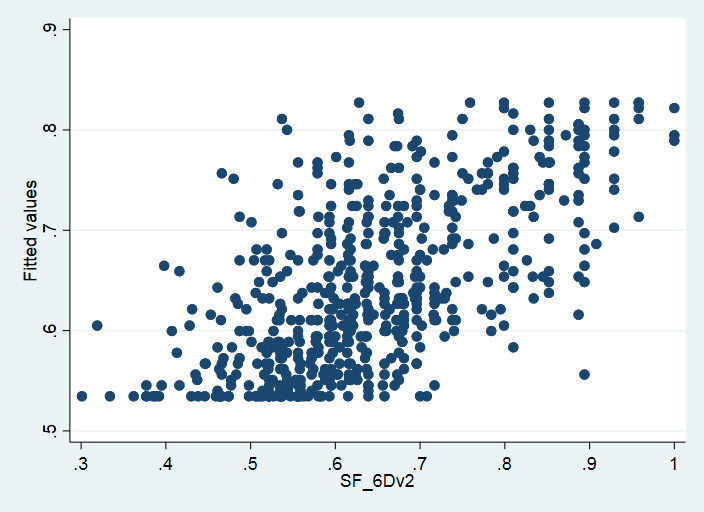


Observed SF-6D vs SF-6D estimated using OLS A
